# Bio-Membrane Internalization Mechanisms of Arginine-Rich Cell-Penetrating Peptides in Various Species

**DOI:** 10.3390/membranes12010088

**Published:** 2022-01-13

**Authors:** Betty Revon Liu, Shiow-Her Chiou, Yue-Wern Huang, Han-Jung Lee

**Affiliations:** 1 Department of Laboratory Medicine and Biotechnology, Collage of Medicine, Tzu Chi University, Hualien 970374, Taiwan; 2 Graduate Institute of Microbiology and Public Health, College of Veterinary Medicine, National Chung Hsing University, Taichung 402204, Taiwan; shchiou@dragon.nchu.edu.tw; 3 Department of Biological Sciences, College of Arts, Sciences, and Business, Missouri University of Science and Technology, Rolla, MO 65409, USA; huangy@mst.edu; 4 Department of Natural Resources and Environmental Studies, College of Environmental Studies, National Dong Hwa University, Hualien 974301, Taiwan

**Keywords:** cell-penetrating peptides, protein transduction, direct membrane translocation, endocytosis

## Abstract

Recently, membrane-active peptides or proteins that include antimicrobial peptides (AMPs), cytolytic proteins, and cell-penetrating peptides (CPPs) have attracted attention due to their potential applications in the biomedical field. Among them, CPPs have been regarded as a potent drug/molecules delivery system. Various cargoes, such as DNAs, RNAs, bioactive proteins/peptides, nanoparticles and drugs, can be carried by CPPs and delivered into cells in either covalent or noncovalent manners. Here, we focused on four arginine-rich CPPs and reviewed the mechanisms that these CPPs used for intracellular uptake across cellular plasma membranes. The varying transduction efficiencies of them alone or with cargoes were discussed, and the membrane permeability was also expounded for CPP/cargoes delivery in various species. Direct membrane translocation (penetration) and endocytosis are two principal mechanisms for arginine-rich CPPs mediated cargo delivery. Furthermore, the amino acid sequence is the primary key factor that determines the cellular internalization mechanism. Importantly, the non-cytotoxic nature and the wide applicability make CPPs a trending tool for cellular delivery.

## 1. Introduction

The plasma membrane is a natural barrier that separates intracellular components from the external environment and keeps the balance of osmotic pressure. However, membrane-active peptides/proteins disrupt and modulate the integrity of the cell membrane. The membrane-active peptides are a term for the peptides that are able to interact with lipid-bilayer membranes to cause leakage as well as damage on cell membranes or to transport exogenous molecules into cells [1,2,3]. Antimicrobial peptides (AMPs), cytosolic peptides and cell-penetrating peptides (CPPs) are three major types of membrane-active peptides [1,4,5]. Here, we focus on CPPs, which are highly applied in biomedical therapeutics and regarded as a potent drug delivery system [6].

## 2. Cell-Penetrating Peptides (CPPs)

Numerous CPPs from natural materials or artificial synthetics were explored in recent decades [6]. The first CPP, a short peptide containing 11 cationic residues, is referred to as the protein transduction domain (PTD). This PTD was initially identified from the Tat (*trans*-activator of transcription) protein of the human immunodeficiency virus type 1 (HIV-1) [7,8]. Since then, various CPPs derived from the PTD of the Tat were reported [9]. Up to now, 1855 CPPs have been defined in CPPsite 2.0, a manually curated database of CPPs [10,11]. Among these 1855 CPPs, 1753 CPPs belong to linear types, whereas 102 CPPs are cyclic peptides. According to the peptide nature, CPPs were categorized into three major groups: protein derived, synthetic, and chimeric peptides [11]. However, according to their chemical and physical properties, most studies have classified CPPs into cationic, amphipathic, and hydrophobic groups [6]. Both AMPs and CPPs are cationic peptides [6,12]. One of the differences between AMPs and CPPs is the lengths of amino acid sequences. The length of AMPs is between 10 and 60 residues and the average number of amino acid residues is 33.26 [12]. Most CPPs contain less than 30 amino acids, but the shortest CPP is a peptide consisting of five residues (L5a, see Appendix A, Table A1) [13,14]. These CPPs can not only enter cells by themselves but can also take cargoes with them to cross cell membranes. The cargoes include proteins/peptides, nucleic acids, peptide nucleic acids (PNA), fluorophores, nanoparticles, drugs and small molecules [6,11]. The link between CPPs and cargoes can be covalent [15,16] or noncovalent [17,18,19,20,21,22,23,24,25,26,27,28,29,30,31,32,33,34], as well as a combination of both (called covalent and noncovalent protein transductions; CNPT) [35,36]. In order to increase the transduction efficiency and stability of the cargoes and to decrease the rate of biodegradability, various strategies of modifications on CPPs were deployed. They include, but are not limited to, the use of D-form amino acid replacements on β or γ sides [37], *cis*- or *trans*-γ-amino acid modification on branches [38], and non-standard amino acid substitutions [39]. However, various modifications of the primary sequences of CPPs, types of cargoes for delivery, combination manners of cargoes, and even concentrations of CPPs were also important factors in contributing to alternative entry routes into cells [27,30,31,34,38,40].

## 3. Mechanisms of Cellular Internalization

**Figure 1:**
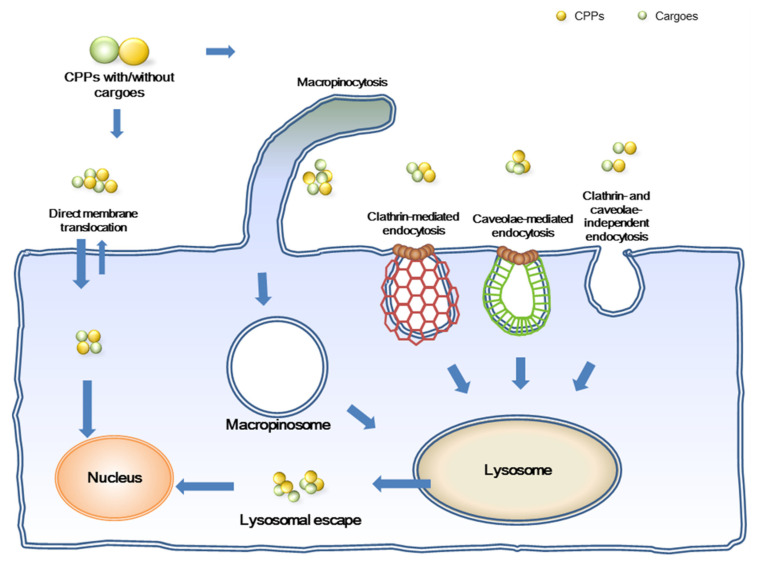
Different cellular entry routes for either cell-penetrating peptides (CPPs) alone or CPP/cargo complexes. Direct membrane translocation and endocytosis are two major routes which have been proposed. Endocytic pathways are further divided into four categories: macropinocytosis, clathrin-mediated endocytosis, caveolae-mediated endocytosis, and clathrin- and caveolae-independent endocytosis.

The understanding of the mechanisms of intracellular uptake for CPPs remains understudied. For example, early research proposed that the Tat protein engaged direct penetration to enter cells [41]. However, subsequent studies have suggested that both direct membrane translocation and endocytosis are employed by the Tat to enter cells [42,43]. To date, the proposed mechanisms for CPPs’ entry into cells are divided into two major categories: energy-dependent endocytosis and energy-independent direct membrane translocation (Figure 1) [44]. Endocytosis of CPPs may involve more than one of the following subtypes: clathrin-mediated endocytosis, caveolae-mediated endocytosis, clathrin- and caveolae-independent endocytosis, and macropinocytosis [44,45]. Macropinocytosis is a kind of F-actin driven lipid raft, which is irrelevant to receptors or any other proteins (Figure 1). It belongs to a non-classical endocytosis [44,45,46]. Both clathrin-mediated and caveolae-mediated endocytosis appertain to dynamin-dependent pathways, while GTPase is involved in all subtypes of endocytosis [45,47,48]. It is arduous to predict the cellular internalization mechanisms of CPPs simply based on their physical and chemical properties. Previous studies indicated that most amphipathic CPPs, such as VP22, KALA and GALA, enter cells via the energy-dependent endocytosis [43]. However, two CPPs, Pep-1 and MPG, which also belong to amphipathic CPP, use direct penetrations for cellular entry [43]. Endocytosis and direct membrane translocation have been proposed as the two major mechanisms used for CPP internalization. Studies were also conducted on several polyarginines with regard to their induction and shifting between different uptake mechanisms under various concentrations of CPPs, cargoes, or cell lines [44,49]. Therefore, in addition to the charge, some critical domains in CPPs were considered as the primary key to determine cellular entry routes. Cationic polyarginines containing many guanidinium groups were regarded as specific CPPs, and the term “arginine-magic” was coined [50,51]. In this communication, we focus on four types of arginine-rich CPPs. Nona-arginines were the backbone of peptide sequences as different modifications or domains were appended on the backbones. We illustrate the cargoes that four arginine-rich CPPs delivered and the cellular internalization mechanisms they used in the next subsections.

### 3.1. Synthetic Nona-Arginine (SR9)

Since the first CPP, the PTD of the Tat, was found, researchers have discovered that cationic amino acids are critical factors to increase protein transduction efficiency [41,52]. Moreover, arginine plays a more important role than lysine in affecting the plasma membrane permeability [52]. Among polyarginine peptides, octa-arginine and nona-arginine were the most commonly used CPPs in cargo deliveries [34,53]. The synthetic SR9 CPPs, containing only nine arginines without any modifications, were able to carry different cargoes, including plasmid DNA, proteins and nanoparticles, and to internalize cell membranes in various species (Table 1). However, properties of cargoes might affect the choice of entry routes. SR9 CPPs enter large unilamellar vesicles (LUVs), artificial plasma-membraned vesicles, via lipid raft inducing (Table 1) [54], while they carry plasmid DNAs to plant tissues through macropinocytosis [19]. The latter intracellular uptake mechanism was also observed in SR9/protein delivery into A549 cells, plant epidermal cells, and mouse skin cells, as well as in SR9/nanoparticle delivery into prokaryotes (Table 1) [17,22,26]. Interestingly, only SR9/quantum dot (QD) complexes employed multiple pathways for entry into mammalian A549 cancer cells [27,33,34]. In summary, both cargoes and entrance targets are important factors influencing the cellular internalization mechanisms of CPPs.

### 3.2. Histidine-Rich Nona-Arginine (HR9)

HR9 CPPs contain extra-modifications with one cysteine and five histidines at both N- and C-termini of the backbone SR9 sequence (Appendix A, Table A1) [27]. Imidazole side chains on histidines possess additional positive charges and may serve as the hydrogen donor or acceptor [57]. A higher zeta potential of HR9 allows for a higher degree of cell membrane disturbance and compactions of HR9/DNA nano-complexes [27,33,55,57,58]. Similar to SR9, HR9 CPPs are able to form complexes with plasmid DNAs, proteins and nanoparticles, and deliver them into mammalian cells, insect cells, paramecia, rotifers and mice (Table 1) [20,21,25,27,29,55,56]. Unlike SR9, different cargoes and entry species play no role in cellular internalization mechanisms of HR9. HR9 CPPs form complexes with either plasmid DNAs or fluorescent QDs and enter A549 cells by direct membrane translocation [25,27,29,33]. Zhang et al. designed a histidine- and arginine-rich CPP, NP1 (see Appendix A, Table A1) to deliver anticancer agent ellipticine and found that these complexes crossed the cell membrane via direct membrane translocation [57]. Although NP1 is composed of the lipid-like domain, HR9 and NP1 CPPs are all rich in histidine and arginine residues, suggesting that direct membrane translocation may rely on sufficient imidazole and guanidinium groups.

### 3.3. Pas Nona-Arginine (PR9)

Endocytosis may lead to cargo trapping and degradations in lysosome. Modifications of the SR9 CPPs with lysosomal escape signals become an option when cargoes are delivered by SR9 into cells via either macropinocytosis or multiple endocytic pathways (Table 1). An inverted and cleft sequence (FFLIPKG), adopted from a lysosomal aspartyl protease, cathepsin D, was chosen for the purpose of lysosomal escape, and it was named as a penetration accelerating sequence (Pas) [59]. PR9 CPPs composed of the SR9 backbone, and a Pas at the N-terminus are able to deliver plasmid DNAs and nanoparticles into A549 cells, Sf9 cells, and paramecia via classical endocytosis (Table 1) [20,21,25,27,28,31,32,33]. Although nanoparticles delivered by PR9 were co-localized with early endosomes and lysosomes at the early stage of intracellular trafficking, lysosomal escape and nuclear accumulation of these fluorescent nanoparticles were observed at the late period of delivery [31,32,33]. Dual Pas and more arginine addition formed a new CPP, known as the Pas2r12(for primary sequence see Appendix A, Table A1) [60,61]. This CPP can deliver large molecules, such as enhanced green fluorescent proteins and immunoglobulin G, through caveolae-mediated endocytosis, and enhance the release of cargoes to cytosol [60,61]. The ways in which Pas-CPPs and cargoes interact do not change the entry routes. Glucagon-like peptide-2 fused with Pas-octa-arginine by covalent bonds can enhance cellular uptake in A549 and MDCK cells via macropinocytosis, as well as translocate to mouse brains via intranasal administration [62]. Lysosomal escape and nuclear localization have also been observed in this Pas-containing arginine-rich CPP [62]. These studies inferred that the critical domain—Pas—plays the key role in endocytic entry route and lysosomal escape. The study by Takayama et al. has further supported this hypothesis. In their study, the Pas domain was replaced by four phenylalanines, and this newly designed F4R8 (primary sequence in Appendix A, Table A1) increased hydrophobicity, thus promoting membrane translocations [63].

### 3.4. INF7 Fusion Nona-Arginine (IR9)

Another endosomolytic peptide—INF7—derived from the influenza hemagglutinin peptide HA2, was fused with SR9 to enhance endosomal escape and cytosolic release [30]. In another study, various CPPs, such as the Tat and Penetratin, were also fused with either HA2 or INF7 to compare the membrane lytic properties [64]. INF7 fused with SR9 (called IR9) enters A549 cells by macropinocytosis, but switches to classical endocytosis when IR9 carries plasmid DNAs or nanoparticles [30]. IR9 can also enter alone or deliver DNAs or QDs as cargoes in rotifers [29]. While the INF7 domain in IR9 triggers membrane insertion using highly conserved hydrophobic residues, the polyarginine domain in IR9 executes cellular internalization [65]. Various amino acids in INF7-Tat were replaced for pH sensitivity studies, and the replacement of glutamic acid at the 6th position of INF7 (A→E) has enhanced lytic potency at lysosomal pH with marvelous pH selectivity [64]. This suggested that the glutamic acid replacement in INF7-CPPs only alters acidic lysosomal membrane without perturbing the cell membrane and, consequently, releases bioactive molecules into cytosol without any cellular injuries. Interestingly, similar to PR9, IR9 is able to enter cells via endocytosis and escape from lysosome [31,32,64]. INF7-CPPs are able to alter secondary structures in pH 5.5 and pH 7.0, while PR9 can maintain the same secondary structures in both pH conditions [31,64]. Many factors, including INF7 positions at either the N- or C-terminus of CPPs, ratios between INF7-CPPs and cargoes, and the types of cargoes, can affect the cellular uptake efficiencies [30,66,67]. Collectively, each variable entails unique properties of INF7-infused CPPs, and more extensive studies are required to identify key variables that are associated with uptake mechanisms and transduction efficiencies.

## 4. Evidence of Cellular Internalization

In this communication, we reveal various cargo-delivery capacities and entry mechanisms of four arginine-rich CPPs in various species (Table 1). Although types of cargoes and penetrating targets influence entry mechanisms, primary sequences play fundamental roles in cellular internalization. Among these four arginine-rich CPPs, SR9 is the backbone of the other CPPs (Table 2). The histidine-rich domains added at both ends of SR9 provide more positive charges, while reverted cathepsin D and INF7 domains give rise to membrane disturbance and lysosome escapes. Some peptide properties, such as net charges, hydrophilicities, hydrophobicities, and theoretical pI, were altered due to these modifications.

Correlations among the structure, properties, mechanism and transduction efficiency of CPPs are a sophisticated issue due to plenty of variables, such as the types and characteristics of cargoes, targets and primary sequences, as well as the chemical structure of CPPs. For example, arginine-rich CPPs usually formed complexes with cargoes in noncovalent manners because of positive/negative charged electrostatic interactions [68]. However, Kamei et al. illustrated that only three cargoes with different pI can be delivered by octa-arginine (R8), while the other 13 peptide drugs cannot [69]. The pI of cargoes and the pH value in the environment are irrelevant to cellular internalization efficiencies. In contrast, the chemical structures, such as L- and D-form, affect cellular internalization efficiencies [70,71]. D-form CPPs are resistant to enzymatic digestions and possess higher stability, which creases cellular internalization [70,71]. Primary sequences of CPPs alter not only cellular internalization efficiencies but also entry mechanisms. While different modified CPPs complexed with or without QDs as the variants, significant differences in cellular internalization efficiencies were observed [25,27,29,33]. The highest efficiency was observed in HR9/QD complexes, while the poor transduction efficiency was displayed in IR9/QD at the same combination ratio of 60 [33]. The INF7 domain in IR9 contributes the highest scale of hydrophobicity and negative charges (Table 2). Therefore, added domains on the SR9 backbone play an important role in determining CPP primary sequences and their transduction efficiencies. Peptide lengths also define the protein transduction efficiencies [70]. The highest insulin absorption is enhanced by R8, instead of 6 or 10 arginine residues [70].

## 5. Conclusions

CPPs have been recognized as a promising tool for intracellular deliveries of bioactive molecules due to their high cellular internalization efficiency. Arginine-rich CPPs are the most common membrane active peptides in biomedical applications. We described unique properties of four arginine-rich CPPs and their capability to internalize a variety of cargoes using different uptake mechanisms. By manipulating their structures, lysosomal escape can be achieved and higher efficiency can be accomplished. A greater understanding of correlations among structures, properties, mechanisms and transduction efficiencies can certainly facilitate the design of more effective CPPs for broader applications in the future.

## Figures and Tables

**Table 1:** Primary sequences of four arginine-rich CPPs and their cellular internalization mechanisms.

CPPs	Peptide Sequences	Cargoes	Entrance Targets	Mechanisms	References
SR9	RRRRRRRRR	—	artificial large unilamellar vesicles (LUVs) in 100 nm diameter	lipid raft	[54]
		plasmid DNAs	plant tissues	macropinocytosis	[19]
			A549 cells, Sf9 cells, paramecia	unknown	[20,21,23,24,28]
		proteins	A549 cells, plant epidermal cells, mouse skin cells	macropinocytosis	[17,22]
		nanoparticles	A549 cells	multiple pathways	[33,34]
			prokaryotes	macropinocytosis	[26]
HR9	CHHHHHRRRRRRRRRHHHHHC	—	A549 cells, rotifers, paramecia	unknown	[21,28,29]
		plasmid DNAs	A549 cells	direct membrane translocation	[29]
			HEK293T cells, Sf9 cells, rotifers, paramecia, mice	unknown	[20,21,29,55,56]
		proteins	rotifers	unknown	[29]
		nanoparticles	A549 cells	direct membrane translocation	[25,27,33]
			rotifers	unknown	[29]
PR9	FFLIPKGRRRRRRRRR	plasmid DNAs	A549 cells, Sf9 cells, paramecia	unknown	[20,21,28,31]
		nanoparticles	A549 cells	classical endocytosis	[25,27,31,32,33]
IR9	GLFEAIEGFIENGWEGMIDGWYGRRRRRRRRR	—	A549 cells	macropinocytosis	[30]
			rotifers	unknown	[29]
		plasmid DNAs	A549 cells	classical endocytosis	[30]
			rotifers	unknown	[29]
		proteins	rotifers	unknown	[29]
		nanoparticles	A549 cells	classical endocytosis	[30]
			rotifers	unknown	[29]

**Table 2:** The properties of four arginine-rich CPPs and their additional modified domains.

CPP	Additional Modified Domain	Net Charge at pH 7.0 ^1^		Hydrophilicity ^1^		Hydrophobicity ^2^		pI ^2^	
		Full Sequence	Domain	Full Sequence	Domain	Full Sequence	Domain	Full Sequence	Domain
SR9	—	+9.0	—	3.0	—	0.77	—	13.4	—
HR9	polyhistidine	+9.82	+0.82	0.95	−0.58	−25.32	−6.34	12.8	7.5
PR9	reverted cathepsin D	+9.95	+1.0	1.2	−0.8	19.74	31.52	13.0	10.1
IR9	INF7 domain	+4.0	−5.0	0.6	−0.34	52.25	65.73	11.9	2.8

^1^ Bachem Peptide Calculator. ^2^ Thermo Fisher Scientific Peptide Analyzing Tool.

## Data Availability

Data available upon request from the corresponding authors.

## Appendix A

**Table A1:** Primary Sequences of cell-penetrating peptides.

CPP	Primary Sequence
F4R8	FFFFGRRRRRRRRGC
HR9	CHHHHHRRRRRRRRRHHHHHC
IR9	GLFEAIEGFIENGWEGMIDGWYGRRRRRRRRR
L5a	RRWQW
NP1	stearyl-HHHHHHHHHHHHHHHHRRRRRRRR-NH_2_
Pas2r12	FFLIGFFLIGRRRRRRRRRRRR
PR9	FFLIPKGRRRRRRRRR
SR9	RRRRRRRRR
